# *Musa dulongjiangensis* (Musaceae), a new species from Yunnan, southwest China

**DOI:** 10.3897/phytokeys.280.196585

**Published:** 2026-10-14

**Authors:** Hu-Biao Yang, Qing-Long Wang, Yun-Yuan Wu, Jin-Hua Chen, Peng Han, Ming-Zhong Huang, Lang-Xing Yuan, Jia-Bao Wang

**Affiliations:** 1 Tropical Crops Genetic Resources Institute, Chinese Academy of Tropical Agricultural Sciences, Danzhou, Hainan 571737, China Tropical Crops Genetic Resources Institute, Chinese Academy of Tropical Agricultural Sciences Danzhou China

**Keywords:** *
Musa
*, *Musa dulongjiangensis*, new species, taxonomy

## Abstract

*Musa dulongjiangensis* (Musaceae), a new species of the genus *Musa* endemic to Yunnan, southwestern China, is described and illustrated in this study. This new species is morphologically similar to *M. puspanjaliae*, but can be clearly distinguished by a combination of morphological characters. Its female bud bracts are dark purple externally and purple internally, with 6–7 basal female flowers arranged in two rows; the base of the compound tepal bears distinct and dense red stripes, the ovary is pale green and glabrous. The male bud bracts are consistent in color with female bracts, dark purple externally and purple internally, and are non-revolute before abscission. Each bract bears approximately twenty male flowers arranged in two rows, with pinkish-purple anther lobes. The fruit bunch is lax, consisting of upward-curving fruits. Typically, each bunch possesses eight hands, with 6–7 fruits per hand, and the pedicels are obviously developed.

## Introduction

In Species Plantarum (1753), Carolus Linnaeus established the genus *Musa*, and by describing *Musa paradisiaca* became the first to assign scientific nomenclature to bananas while also establishing the modern botanical nomenclature still widely used today (Häkkinen 2013). Over the past 200 years, the genus *Musa* has undergone multiple taxonomic revisions. In 1893, the genus was divided into three subgenera (Baker 1893): *Eumusa*, *Rhodochlamys*, and *Physocaulis*. The most transformative contribution came from Cheesman, who divided the genus into four sections: *Australimusa* (2n = 20), *Callimusa* (2n = 20), *Musa* (2n = 22) and *Rhodochlamys* (2n = 22) based on chromosome numbers and morphological characters (Cheesman 1947, 1948, 1949). However, many new studies have posed a challenge to traditional views, including genetic similarities shown by AFLP markers and the discovery of species with unusual chromosome numbers, and molecular phylogeny does not support the recognition of four monophyletic sections etc. (Li et al. 2010; Liu et al. 2010). In 2013, Häkkinen published a landmark taxonomic revision in which he restructured the genus into only two sections based on molecular phylogenetic evidence: sect. *Musa* (including all former *Eumusa* and *Rhodochlamys* species with x = 11) and sect. *Callimusa* (including all former *Australimusa*, *Callimusa*, and *Ingentimusa* species with x = 10, x = 9, and x = 7), thus formally consolidating the genus and reflecting its evolutionary history more accurately (Häkkinen 2013).

Currently, according to statistical data from POWO (Plants of the World Online), there are 86 accepted species of the genus *Musa* worldwide, distributed across tropical and subtropical regions of Asia, the Pacific, and Australia (https://powo.science.kew.org/). Southern and southwestern China are also important regions for the distribution of wild *Musa* species. Among these, Yunnan stands out as the most species-rich province, hosting a number of endemic or regionally restricted wild *Musa* (Liu et al. 2002; Häkkinen and Wang 2007, 2008a, 2008b; Häkkinen 2009; Chen et al. 2014; Sachter-Smith 2023). Based on the literature as well as the authors’ recent surveys and collections, at least the following 30 wild Musa taxa (including species and varieties) currently occur naturally in China, namely: *M. acuminata*, *M. balbisiana*, *M. basjoo*, *M. huangbaoia*, *M. itinerans* (M. itinerans var. annamica, M. itinerans var. xishuangbannaensis, M. itinerans var. lechangensis, M. itinerans var. hainanensis, M. itinerans var. formorson, M. itinerans var. kavanlanensis, M. itinerans var. chiumei, M. itinerans var. chinensis, M. itinerans var. guangdongensis), *M. splendida*, *M. yunnanensis* (M. yunnanensis var. yunnanensis, M. yunnanensis var. caii, M. yunnanensis var. yongpingensis and M. yunnanensis var. jingdongensis), *M. zaifui*, *M. chunii*, *M. ruiliensis*, *M. rubinea*, *M. nagensium*, *M. sanguinea*, *M. cheesmanii*, *M. aurantiaca*, *M. markkuana*, *M. exotica* and *M. puspanjaliae* (Wu and Kress 2000; Liu et al. 2002; Häkkinen and Wang 2007, 2008a, 2008b; Häkkinen and Teo 2008; Häkkinen and Väre 2008; Häkkinen et al. 2008; Häkkinen 2009; Chiu et al. 2011; Chen et al. 2014; Sachter-Smith 2023).

A survey of Musaceae plant resources was conducted in the Dulong River area (Dulongjiang Township, Gongshan County, Yunnan Province) between 2023 and 2025. A distinctive population of wild *Musa* was encountered that could not be assigned to any known species of the genus. After detailed morphological observation and comparison, this species is morphologically most similar to *M. puspanjaliae* in having a robust pseudo-stem, a pendent inflorescence position, leaves that completely die off and become deciduous before fruit maturity, larger seeds (flat and irregular) with a top width of 1 cm or more, and other characteristics. However, the two species differ distinctly in multiple morphological traits, including inflorescence, female bud, bracts, basal female flowers, compound tepals, ovary, male bud, male flowers, fruit bunches, fruit finger orientation and fruits size, among others. Consequently, it is confirmed to be an independent new species.

## Materials and methods

The new species is described based on field observations of fresh materials conducted in its native habitat, the Dulongjiang area of Yunnan Province, and examinations of herbarium specimens preserved in the Herbarium of Tropical Crops Genetic Resources Institute, Chinese Academy of Tropical Agricultural Sciences. Observation of *M. puspanjaliae* was carried out through field observations and on *ex situ* conserved plants at the Hainan Tropical Botanical Garden, and image collection of various detailed parts was completed. The observation of plant parts, including the plant, inflorescence, bracts, female flowers, compound tepals, free tepals, ovary, and fruits, was completed using a Nikon D810 camera. The studied specimens are kept in the Herbarium of the Tropical Crops Genetic Resources Institute of the Chinese Academy of Tropical Agricultural Sciences (ATCH).

## Taxonomic treatment

### Musa dulongjiangensis

Taxon classificationPlantaeZingiberalesMusaceae

H.B.Yang sp. nov.

0F3300F0-E7E1-58B1-8F1F-7C20018C294B

urn:lsid:ipni.org:names:77412362-1

Fig. 1

#### Diagnosis.

*M. dulongjiangensis* is similar in morphology to *M. puspanjaliae*, but they differ significantly in the following morphological features. Sterile bracts 2 (vs. 4), female bud bracts dark purple externally (vs. greenish-yellow or purple), female flowers, 6–7 flowered in two rows (vs. 13–16 flowered in two rows), ovary pale green, glabrous (vs. cream, slightly pubescent), compound tepal 3 cm long, base with distinct and dense red stripes (vs. 5 cm long, without red stripes), free tepal broadly ovate (vs. oblong ovate to triangular), male bud bracts unrevolute before falling (vs. revolute before falling), male buds uncovered with persistent dry old bracts (vs. covered with persistent dry old bracts), male buds incompletely fall off (vs. fall off completely), male flowers on average 20 per bract in 2 rows (vs. 22 per bracts in 2 rows), anthers lobe pinkish purple (vs. creamy to pale pink); fruit bunch lax, with 8 hands and 6–7 fruits per hand, in 2 rows (vs. very compact, with 8 hands; fruits 15 per hand, in 2 rows), individual fruit curved upward (vs. straight), fruits with short pedicel (vs. sessile).

**Figure 1.**
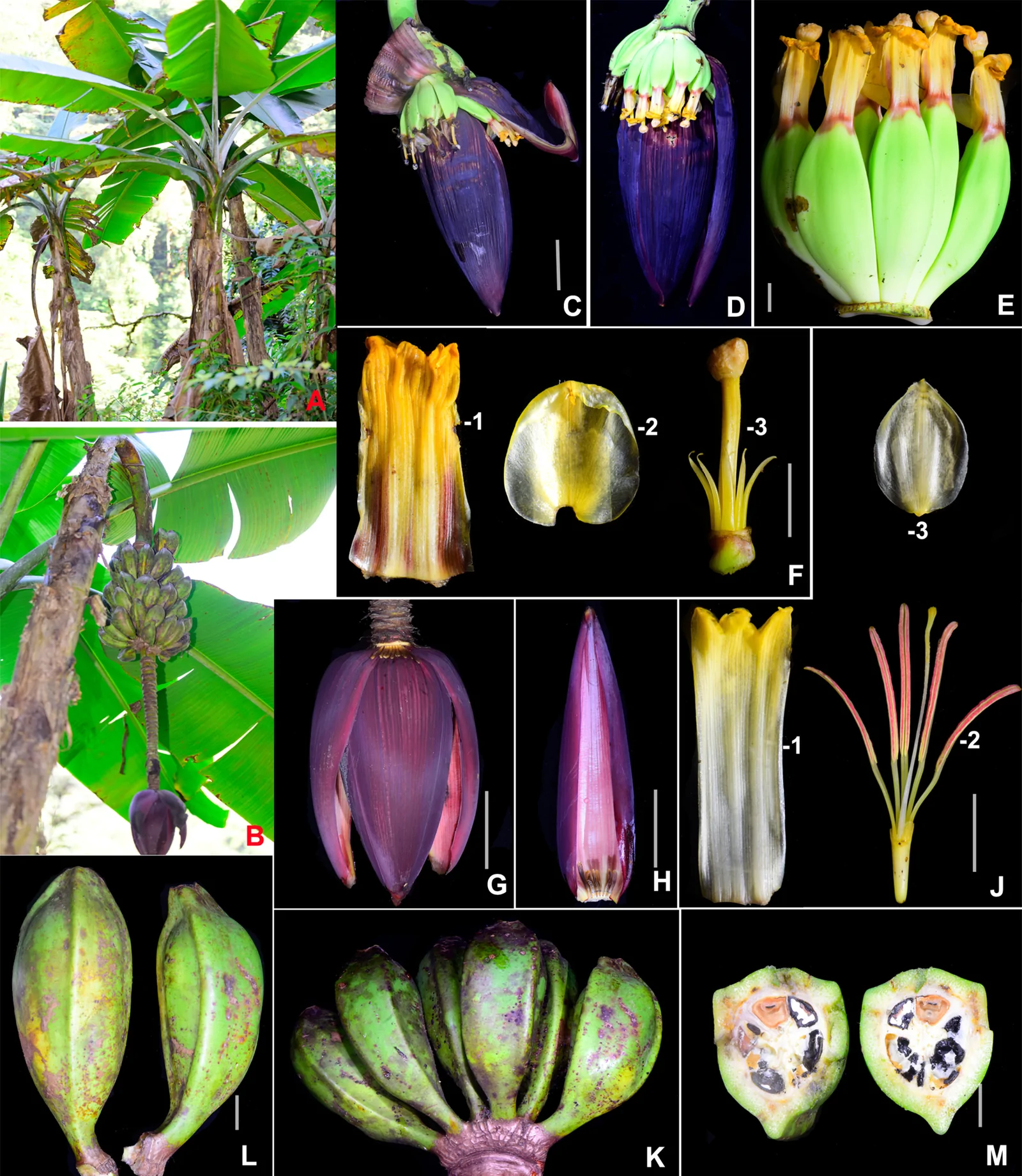
*Musa dulongjiangensis* H.B.Yang, sp. nov. **A**. Plants in the habitat; **B**. Mature fruiting bunch; **C**. Inflorescence with flowers; **D**. Internally of female bud bracts; **E**. Basal female flowers and ovaries; **F**. (-1, -2, -3) compound tepal, free tepal, sterile stamen and stigma of female flower; **G**. Male bud; **H**. Internally of male bud bract; **J**. (-1, -2, -3) compound tepal, free tepal and stamen of male flower; **K**. Hand of fruits; **L**. Individual fruit and curved; **M**. Ovules in 2 rows per locule. Scale bars: 5 cm (**C, G, H**); 1 cm (**E, F, J, L, M**).

Morphological feature photographs of the closely related species *M. puspanjaliae* are shown in Fig. 2. Field observations and photographic documentation of *M. puspanjaliae* were carried out in its natural habitat in Mêdog County, Xizang. This species is abundant in river valleys at elevations of 1000 m and below in Mêdog. Surveys confirm that *M. dulongjiangensis* is not distributed in this area. *M. dulongjiangensis* also occurs at approximately 1000 m above sea level. However, neither literature records nor our field surveys have documented the presence of *M. puspanjaliae* in the Dulongjiang area of Yunnan Province.

**Figure 2.**
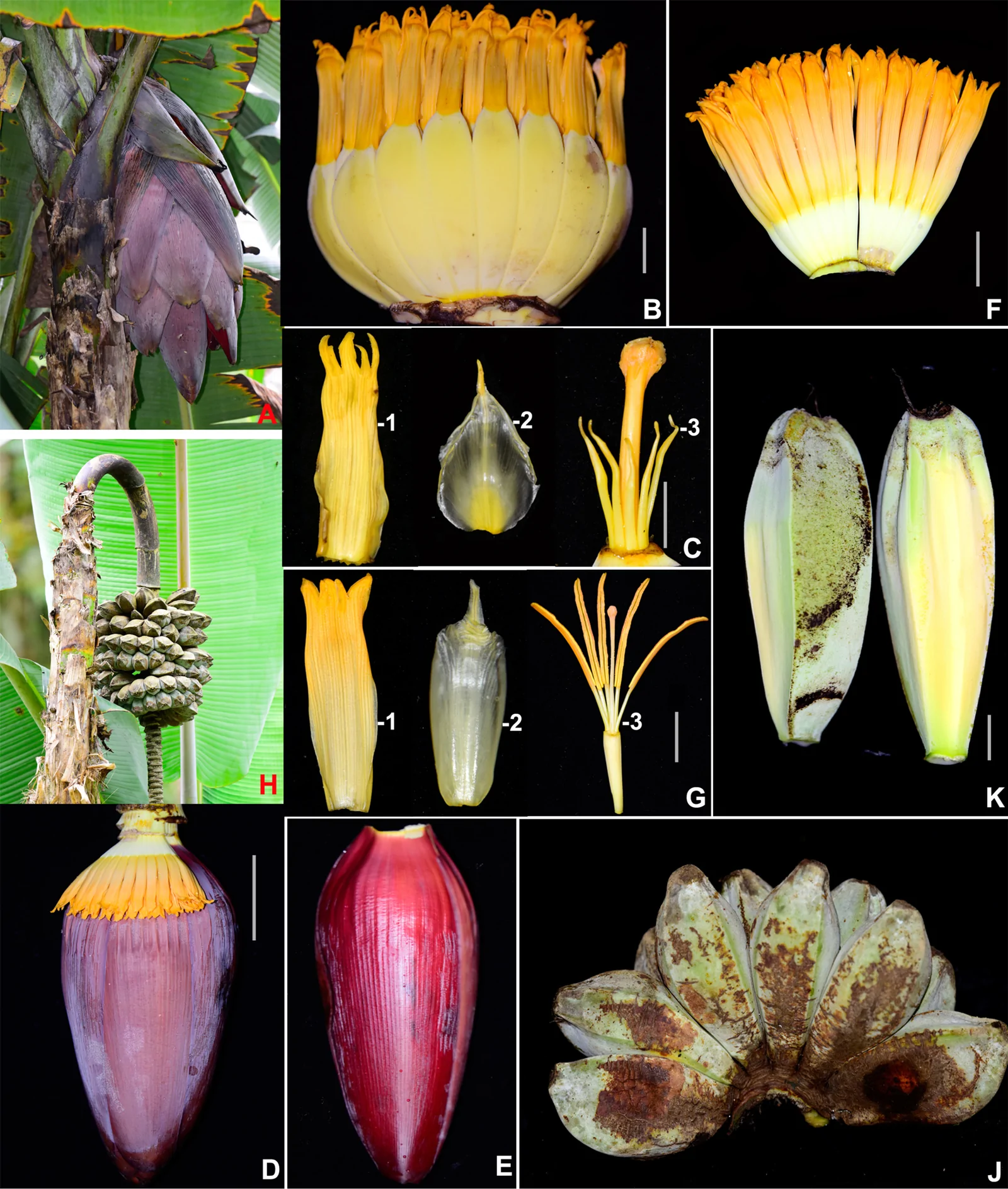
*Musa puspanjaliae*. **A**. Inflorescence; **B**. Basal female flowers and ovaries; **C**. (-1, -2, -3) compound tepal, free tepal, sterile stamen and stigma of female flower; **D**. Male bud; **E**. Internally of male bud bract; **F**. Male flowers; **G**. (-1, -2, -3) compound tepal, free tepal and stamen of male flower; **H**. Mature fruiting bunch; **J**. Hand of fruits; **K**. Individual fruit and not curved. Scale bars: 5 cm (**D**); 1 cm (**B, C, F, G, K**).

#### Type.

China. • Yunnan Province, Nujiang Prefecture, Gongshan County, Dulongjiang Township, growing on the forested slopes along the banks of the Dulong River, 27°40'N, 98°16'E, 1194 m a.s.l., 12^th^ December 2024, *H.B.YANG et al. 202412073* (holotype: ATCH; isotype: ATCH).

#### Description.

Plants robust, clumped, suckering freely, with up to 2–4 suckers, vertical, 4–6.5 m tall, ca. 25 cm in diameter at base. Sheaths usually covered with dead remains of old sheaths, not waxy, underlying color green, shiny. Leaves intermediate; petioles ca. 60 cm long, green, waxy, margins curved in petiole canal, winged and undulating; edge brownish at first, later becoming withered, less than 1 cm wide. Lamina up to 200 × 60 cm, oblong-lanceolate, truncate at the apex, green adaxially, medium green abaxially; leaf bases symmetric, both sides rounded and auriculate; midrib dorsally light green and waxy. Inflorescence pendent; peduncle to 35 × 5 cm, sparsely pubescent at an early stage, becoming glabrous later, light green to dark green, with brown spots; sterile bracts 2, deciduous at the opening of the first female flowers. female bud lanceolate, ca. 40 cm long, 15 cm wide, bracts dark purple externally and waxy, dark purple or purple internally, lifting few bracts at a time, revolute before falling; basal flowers female, 6–7 flowered in two rows, each flower ca. 10 cm long; ovary pale green, slightly curved, 3-angled, to 7 cm long and 2 cm wide, glabrous; ovules in 2 rows per locule; compound tepal light orange, base with distinct red spots, 3 cm long, 1.2 cm wide, with 2 keels, its apical portion distinctly 3-lobed; lobes acute, yellow colored; free tepal broadly ovate, 1.7 cm long, 1.6 cm wide, yellow and trans-lucent, apex apiculate; Stamens 5, yellow, incompletely developed, anthers absent, style ca. 2 cm long, yellow, stigma yellow, 4 mm in diameter. Male buds ovate-lanceolate, to 25 cm long and 11 cm wide, whole buds incompletely fall off even on maturity; bracts dark purple externally and slightly waxy, purple internally, unrevolute before falling, uncovered with persistent dry old bracts; male flowers on average 20 per bract in 2 rows, falling with the bract, compound tepal ca. 4 cm, upper part yellow, lower part cream, with thickened keel, ribbed at the dorsal angles, with 5-toothed orange apex, the central lobes smaller than the outer lobes, free tepal ca. 1.5 cm, translucent cream, oval, smooth, apex apiculate; stamens 5, filaments pale yellow, anthers’ lobes pinkish purple, anthers and style exserted, stigma cream; ovary straight, cream color, abortion. Fruit bunch lax, fingers curved upward, with 8 hands and 6–7 fruits per hand on average, in 2 rows; individual fruit 10–13 cm long, 4–5 cm wide, curved, with a pronounced ridge (3-ridged), pedicel ca. 2 cm, glabrous, brown scar-like proliferation at the point of attachment to the rachis, immature peel color green, becoming pale yellowish-green, with irregular light brown spots and a rough texture at maturity. Seeds irregularly flattened, wrinkled, angular, 8–10 mm long and 12–15 mm wide at the top.

#### Phenology.

According to observation, the main flowering period of the *M. dulongjiangensis* is from April to August, and the fruit ripening period is from October to March of the following year.

#### Etymology.

The specific epithet *dulongjiangensis* refers to the type locality, Dulongjiang Township in Gongshan County.

#### Vernacular name.

Dú lóng jiāng bā jiāo (独龙江芭蕉) (Chinese).

#### Distribution and ecology.

The climate in the area where *M. dulongjiangensis* occurs belongs to the subtropical warm and humid type, characterized by abundant rainfall and high humidity throughout the year, with an annual precipitation reaching as high as 2932–4000 mm and relative humidity remaining around 90% year-round. This new species mainly grows on forested slopes at an elevation of about 1100 m along both sides of the Dulong River Valley, preferring semi-shade and high humidity conditions. Common accompanying species of the same family in the community include *M. chunii* and *M. markkuana*.

#### Conservation status.

According to current investigations, *M. dulongjiangensis* is distributed in valley areas below 1300 m elevation along both sides of the Dulong River (Fig. 3). As the elevation decreases along the river, its distribution range becomes relatively larger. The number of individuals within the community is relatively high, and the seeds in its fruits are numerous, which is an advantageous trait for population expansion. Secondly, its distribution area falls within the scope of the Gaoligong Mountain National Nature Reserve. Based on the above information, the overall conservation status of this newly described species is presumably favorable. However, as its distribution extends downstream into Myanmar, we have been unable to carry out extensive surveys. We therefore recommend assessing it as Data Deficient (DD) in accordance with the IUCN Red List Categories and Criteria (IUCN 2024).

**Figure 3.**
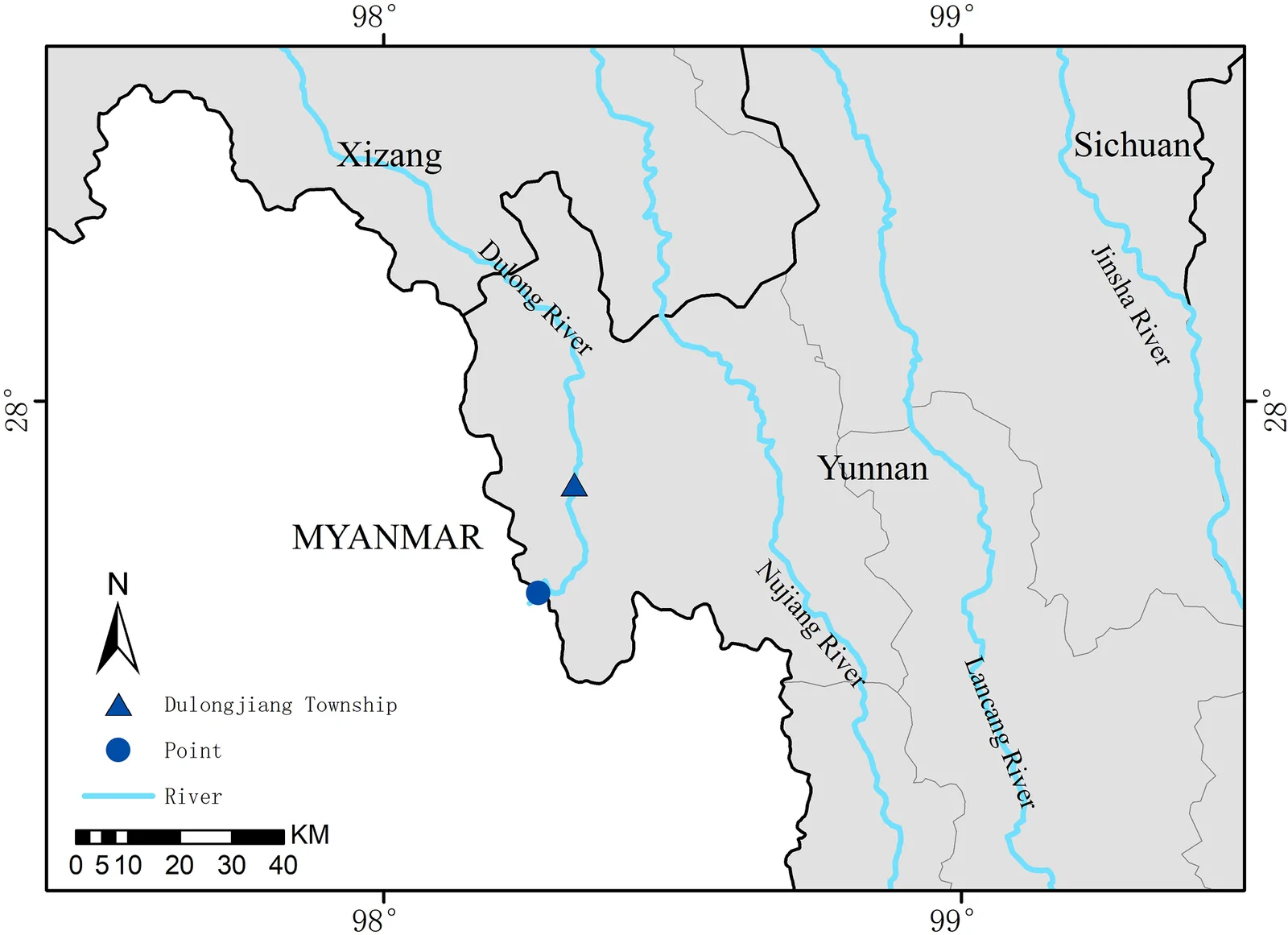
Geographic distribution of *Musa dulongjiangensis*.

## Discussion

The southern slopes of the Himalaya represent a significant center of diversification for the genus *Musa* (Janssens et al. 2016; Jenny et al. 2024). In China, the low-altitude gorge areas of the Dulong River basin and the Yarlung Tsangpo River basin have formed suitable habitats for the growth of wild bananas, thereby promoting lineage diversification and species persistence. Over recent decades, botanical surveys in these regions have led to the continuous discovery of new species or new distribution records. Notable examples include *M. yunnanensis* and M. acuminata var. chinensis (Häkkinen and Wang 2008a, 2008b), *M. chunii* (Häkkinen 2009), as well as *M. siangensis* from the eastern Himalayan arc. The newly discovered *M. dulongjiangensis* is a particularly important addition to the genus *Musa*. The discovery of this species adds to mounting evidence that the northern margin of the genus *Musa*’s natural range harbors far greater diversity than previously thought. These findings have profound conservation implications, as wild *Musa* species are the genetic reservoir for cultivated bananas, offering traits such as disease resistance and stress tolerance. The discovery of *M. dulongjiangensis* underscores the need for continued taxonomic research in the eastern Himalaya, provides a basis for conservation planning, and expands the pool of wild genetic resources for banana breeding (Perrier et al. 2011). Future efforts should include targeted field surveys, molecular phylogenetic studies, and integrated *in situ* and *ex situ* conservation programs.

## Supplementary Material

XML Treatment for Musa dulongjiangensis

## Additional information

### Artificial Intelligence (AI) use

The authors accept full responsibility for the content of the manuscript, including the disclosure of any use of AI.

Regarding the use of AI in the preparation of this manuscript, the authors declare the following: DeepSeek.

**Used for**: Other.

The use of AI is primarily for language translation.

### Author ORCIDs

H.-B. Yang https://orcid.org/0000-0002-7380-8874

Q.-L. Wang https://orcid.org/0000-0002-9834-6944

Y.-Y. Wu https://orcid.org/0009-0007-7858-1211

J.-H. Chen https://orcid.org/0009-0009-8033-4222

P. Han https://orcid.org/0009-0000-1835-485X

M.-Z. Huang https://orcid.org/0000-0002-5403-6450

L.-X. Yuan https://orcid.org/0000-0003-2102-9075

J.-B. Wang https://orcid.org/0009-0004-4833-121X

### Data availability

All of the data that support the findings of this study are available in the main text.
